# Postoperative pain and perioperative outcomes after laparoscopic radical hysterectomy and abdominal radical hysterectomy in patients with early cervical cancer: a randomised controlled trial

**DOI:** 10.1186/1745-6215-14-293

**Published:** 2013-09-12

**Authors:** Luciana Silveira Campos, Leo Francisco Limberger, Airton Tetelbom Stein, Antonio Nocchi Kalil

**Affiliations:** 1 Serviço de Ginecologia do Hospital Nossa Senhora da Conceição, Av Francisco Trein, 596, Bairro Cristo Redentor CEP, Porto Alegre 91350-200, Brazil; 2 Universidade Federal de Ciências da Saúde de Porto Alegre, Brazil, Rua Sarmento Leite, 245 CEP, Porto Alegre 90050-170, Brazil; 3 Serviço de Epidemiologia do Hospital Nossa Senhora da Conceição, Porto Alegre, Brazil, Av Francisco Trein, 596, Bairro Cristo Redentor CEP, Porto Alegre 91350-200, Brazil

**Keywords:** Cervical cancer, Laparoscopy, Radical hysterectomy

## Abstract

**Background:**

Non-randomised studies have suggested that the postoperative complications of (Campos LS, Limberger LF, Stein AT, Kalil AN) laparoscopic radical hysterectomy are similar to those in abdominal radical hysterectomy. However, no study evaluating postoperative pain comparing both techniques has been published thus far. Our objective was to compare pain intensity and other perioperative outcomes between laparoscopic radical hysterectomy (LRH) and abdominal radical hysterectomy (ARH) in early cervical cancer.

**Methods:**

This single centre, randomised, controlled trial enrolled 30 cervical cancer patients who were clinically staged IA2 with lymph vascular invasion and IB according to the FIGO (International Federation of Gynaecology and Obstetrics) classification, and underwent LRH or ARH between late 1999 and early 2004. Postoperative pain, as measured by a 10-point numerical rate scale, was considered the primary endpoint. Postoperative pain was assessed every six hours during a patient’s usual postoperative care. Perioperative outcomes were also registered. Both surgical techniques were executed by the same surgical team. Secondary outcomes included intraoperative and other postoperative surgicopathological factors and 5-year survival rates.

**Results:**

IA2 patients with lymphatic vascular space invasion and IB cervical cancer patients were randomised to either the LRH group (16 patients) or the ARH group (14 patients). Four patients (25%) in the LRH group and 5 patients (36%) in the ARH group presented with transoperative or serious postoperative complications. All of the transoperative complications occurred in the LRH group. The relative risk of presenting with complications was 0.70; CI 95% (0.23–2.11); *P* = 0.694. LRH group mean pain score was significantly lower than ARH after 36 h of observation (*P* = 0.044; mean difference score: 1.42; 95% CI: 0.04–2.80). The survival results will be published elsewhere.

**Conclusions:**

LRH provided lower pain scores after 36 h of observation in this series. The perioperative and serious postoperative complications ratios were comparable between the groups.

**Trial Registration:**

NCT01258413

## Background

Cervical cancer is the second most common cancer among women worldwide, and 83% of cases occur in developing countries [1]. In Brazil, the estimated annual incidence is approximately 19 cases per 100,000 women [2]. Radical hysterectomy with pelvic lymphadenectomy is a (FIGO) International Federation of Gynaecology and Obstetrics recommended treatment for early cervical cancer [3], traditionally performed using the abdominal approach [4].

The advantages of laparoscopic versus the open approach for benign gynaecological diseases reported in the literature include decreased postoperative pain and a shorter hospital stay [5,6]. Some randomised studies have reported an increased operative time [6,7].

Previous studies have shown the feasibility and safety of laparoscopic radical hysterectomy (LRH) [8-10], and non-randomised controlled studies have suggested that LRH has an increased operative time [11-13], a shorter hospital stay [11,12], and fewer postoperative infections [12] compared with the open approach. A histopathological comparison of LRH and abdominal radical hysterectomy (ARH) suggested that they have the same radicality [14]. Preliminary data suggested an equivalent survival rate for the two techniques [13,15].

No randomised trials are available that compare laparoscopic radical hysterectomy with abdominal laparoscopic hysterectomy for treating early cervical cancer. A randomised, prospective international protocol was performed with 740 patients enrolled to evaluate the feasibility, complications, quality of life, and survival in early cervical cancer patients assigned to either abdominal (ARH) or laparoscopic/robotic radical hysterectomies (LRH). Equivalence will be declared if the disease-free survival difference does not exceed 7% at four years [16].

## Methods

The detailed eligibility criteria, recruitment details, endpoints, standardisation of surgical procedures, surgical techniques, sample size calculation and randomisation methods have been published elsewhere [17]. The surgeons that performed the procedures assessed patients in postoperative care during hospital stay and its complications; during the follow-up, the same surgeons evaluated and managed the recurrences. The nurses who took care of the patients and who measured pain as the 5^th^ vital sign during hospital stay were also not blind. Parametrial extension, vaginal cuff, and uterosacral ligament sizes were measured by the leading surgeon in the operative room. Operative time was measured by the study team surgeons. Other surgicopathological factors were assessed by the attending pathologist after tissue processing.

Briefly, this is a single centre, randomised, controlled trial comparing the hysterectomy LRH and ARH procedures. Eligible patients were randomised in either the LRH or ARH groups. The primary endpoint was postoperative pain, as measured by a 10-point numerical rating scale (NRS) during the postoperative period. Pain was assessed every 6 h by the nursing staff during a patient’s usual postoperative care; the nursing staff had been trained at the beginning of the study. One day pre-surgery, they were reminded to ask each patient to score her pain level from 0–10; the nursing staff was unaware of the study objective. The secondary outcomes were intraoperative and postoperative complications, histopathological characteristics, overall survival and disease-free survival; these outcomes were registered prospectively.

### Inclusion criteria

The following inclusion criteria were applied: women ≥18 years who sought treatment for histologically confirmed primary squamous, adenocarcinoma, or adenosquamous cervical cancer that was diagnosed by biopsy or cervical conization and clinically staged according to the FIGO classification of IA2 with IB or II A lymph vascular invasion [3].

### Exclusion criteria

The following exclusion criteria were applied: patients with clinically advanced disease (IIB-IV), previous pelvic or abdominal radiotherapy, pregnancy or clinical diseases that would preclude one or both surgical approaches (pulmonary obstructive disease poorly controlled or contraindicating prolonged Trendelenburg position, severe hip disease precluding the use of the dorsolithotomy position; inadequate bone marrow, renal, or hepatic function); obesity; previous abdominal or pelvic surgery and demographic factors were not considered exclusion criteria.

Each surgery was performed by the same team. The leading surgeon (LFC) performed all of the surgeries, and the other team members were first or second assistants (RK and LSC). The leading surgeon had performed more than one hundred abdominal radical hysterectomies before performing laparoscopic radical hysterectomies. Before the beginning of the study, a surgeon with expertise in abdominal radical hysterectomies standardised the surgical technique by evaluating the digital records of the laparoscopic radical hysterectomies that were performed by the first surgeon. To evaluate oncologic adequacy, the leading surgeon measured the parametrial and vaginal tissue in the operative room before processing the tissue [18].

### Surgical techniques

#### Laparoscopic radical hysterectomy and laparoscopic pelvic lymphadenectomy

Both procedures were performed as previously described [8,9,19]; LRH involves placing a uterine manipulator with chrome place tubing. Access to the abdominal cavity was obtained though insertion of Verres needle and insufflation before placement of a 10-mm trocar. Laparoscopic radical hysterectomy was performed according to Piver III classification for a radical hysterectomy [20]. The vaginal cuff was sutured laparoscopically. Close-suction drainage was placed until the daily drainage fell below 100 mL.

ARH was performed according to the Piver type III classification for a radical hysterectomy [20]. Access to the abdominal cavity was obtained through a vertical midline skin incision.

#### Anaesthesia and postoperative analgesia

The same team of anaesthesiologists performed anaesthesia following a defined protocol: midazolam 15 mg orally one hour before surgery, IV access and standard monitoring. General anaesthesia was induced with fentanyl 3 μg/kg and propofol 2 mg/kg. Orotracheal intubation was facilitated by atracurium 0.5 mg/kg. After intubation, the lungs were ventilated with 50% O_2_, 50% N_2_O, and 2% sevoflurane. At the beginning of the lymphadenectomy, ketoprofen 100 mg and metoclopramide 10 mg were administrated via IV. Before the extubation, dipyrone 15 mg/kg IV, and morphine 0.05 mg/kg SC were administered. Residual neuromuscular blockade was antagonised with neostigmine and atropine when necessary.

Postoperative analgesia: Day 1, diclofenac 75 mg IM BID, dipyrone (15 mg/kg) IV QID and morphine 0.05 mg/kg mg SC every four hours; Day 2, diclofenac 50 mg PO tid and morphine 3 mg SC every four hours (on demand); Day 3, diclofenac 50 mg PO on demand and morphine 3 mg SC every four hours (on demand).

### Adjuvant radiotherapy/chemotherapy

At the discretion of the responsible physician, the histopathological findings were used to determine the need for adjuvant postoperative treatment.

### Postoperative follow-up

All of the patients were evaluated by the study team in the early postoperative period. The long-term follow-up was a minimum of five years by the study team or the patients’ assistant physician. Periodically, contact was made with the assistant physician by the surgeons of the study team to ascertain the patients’ status. All serious complications were documented and managed by the study team.

### Assessing perioperative complications

All of the transoperative and serious postoperative complications were noted. We analysed the proportion of patients presenting with perioperative complications in both groups.

### Sample size calculation

The NRS scale was considered the primary postoperative endpoint. We expected a 55% difference in pain scale intensity between the groups. The sample size was calculated by Epi Info version 6.04b software in a 1:1 sample, and we obtained 30 patients.

### Randomisation

The patients were randomly assigned to groups using a random number table of 180 five-digit numbers generated by an independent author (ATS) who did not participate in patient selection, surgery or follow-up. After informed consent signing and prior to surgery, a random allocation number was determined with a telephone call.

### Statistical analysis

Continuous variables with normal distribution were analysed using Student’s *t-*test for independent samples and described by means and standard deviation, whereas those not consistent with normal distribution were analysed using the Mann-Whitney *U* test and described as medians and percentiles. The discrete variables were compared using Pearson’s *χ*^2^ test, and Fisher’s exact test was used when any cell was <5. The pain scores and intergroups were analysed with an ANOVA for the repeated measures, and the Bonferroni correction was applied as necessary. Statistical significance was set at *P* <0.05. The statistical software package SPSS (Statistical Package for the Social Sciences) version 18.0 was used for all data analysis.

### Ethical considerations

The Ethics Committee of Grupo Hospitalar Conceição approved the study protocol in 1999. This protocol was registered at ClinicalTrials.gov. Written informed consent was obtained from all patients prior to enrolment.

## Results

**Figure 1:**
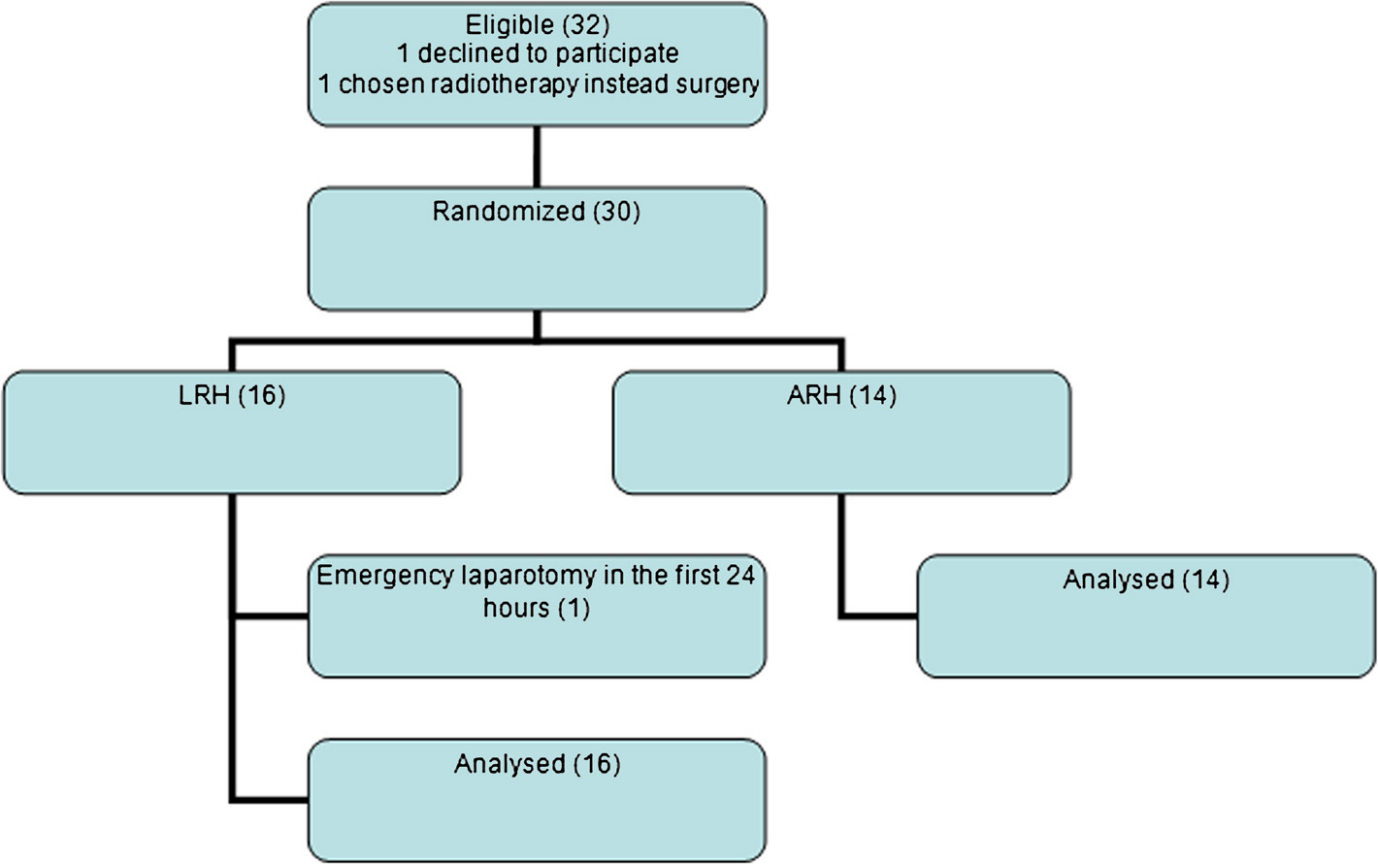
Study flowchart.

From 1999 to 2004, 30 patients were included in this trial and underwent randomisation; 16 were submitted to LRH and 14 to ARH (Figure 1). No conversion to laparotomy occurred in the LRH group. Table 1 shows some patient characteristics; Table 2 shows the surgicopathological factors. There was no difference in the pelvic lymph node count between the LRH and ARH groups. Parametrial extension was significantly longer for patients submitted to LRH compared to ARH (*P* = 0.07 for the right parametrium and *P* = 0.002 for the left parametrium) (Table 2). There was no difference in operative time between the groups.

**Table 1:** Patient characteristics

**Variables**^**1**^	**ARH (14)**	**LRH (16)**
Age	39.64 ± 6.23	36.19 ± 9.78
Number of pregnancies	3.5 (3.0–4.0)	2.5 (1.5–3.5)*
Smoking	6 (43%)	5 (31%)
Previous conization	6 (43%)	8 (50%)
Previous abdominal or pelvic surgery	5 (36%)	7 (44%)

^1^Described as mean ± standard deviation or median (percentile 25–75), if quantitative. If categorical n (%).

*P <0.05.

**Table 2:** Surgicopathological factors

**Variables**	**ARH (14)**	**LRH (16)**
Squamous	12 (86%)	12 (75%)
Adenocarcinoma	2 (14%)	4 (25%)
LVSI^1^	6 (43%)	4 (25%)
Lymph nodes (number)	19.43 ± 6.35	19.5 ± 8.41
Right parametrial tissue^2^	4.14 ± 1.56	6.44 ± 2.59*
Left parametrial tissue^2^	4.01 ± 1.42	6.33 ± 2.2*
Right uterosacral ligament	2.34 ± 0.91	2.78 ± 1.1
Left uterosacral ligament	2.26 ± 0.97	2.67 ± 1.1
Vaginal cuff	2.41 ± 1.18	3.17 ± 1.28
Operative time	240.36 ± 26.85	264.38 ± 49.29

^1^LVSI: Lymph vascular space invasion; ^2^Measured in cm.

*P <0.05.

Table 3 shows all the transoperative and serious postoperative complications. Some patients presented with more than one complication. There were four transoperative complications, all in the LRH group. Two patients underwent inadvertent cystotomies during bladder dissections; both were recognised intraoperatively and sutured laparoscopically. One patient presented with left renal exclusion three months after surgery and underwent nephrostomy. Ten months after the LRH, she presented with a vesicovaginal fistula and underwent a successful surgical correction. Two years after LRH, the patient underwent a left nephrectomy. The other patient had an otherwise uncomplicated recovery.

**Table 3:** Surgery-related complications

**Complications**	**ARH**	**LRH**	N*
Cystotomy	0	2	2
Bowel injury	0	2	2
Ureterostenosis	1	0	1
Ureterovaginal fistula	0	1	1
Vesicovaginal fistula	0	2	2
Rectovaginal fistula	0	1	1
DVT**/PE	0	1	1
Intra-abdominal infection	3	1	4

*Some patients presented more than one complication.

**Deep venous thrombosis/Pulmonary embolism.

One patient underwent a rectal injury that was sutured vaginally, and on the 14^th^ postoperative day, she presented with a ureterovaginal fistula, which was initially unsuccessfully managed with a JJ catheter. She later underwent a successful correction of the fistula 30 days post-surgery. Another patient presented with a bowel injury unrecognised at the time of surgery and required an additional laparotomy the day after the LRH. An emergency descending colectomy and a colostomy were performed with washing of the cavity. She developed sepsis and deep venous thrombosis, and underwent a vesicovaginal fistula correction during a subsequent hospitalisation. She also presented with a rectovaginal fistula 24 months after LRH. This patient has been disease-free for 9 years and was fitted with a colostomy pouch. In the ARH group, one patient developed ureterostenosis after undergoing pelvic radiation indicated for positive pelvic lymph nodes; three patients group presented with abdominal sepsis and had favourable outcomes; and one was diagnosed with renal exclusion and underwent a nephrectomy.

Four (25%) patients in the LRH group presented with transoperative complications, but none of the patients in the ARH group presented with transoperative complications (Fisher’s exact test = 0.10). Three patients (19%) in the LRH group presented with serious postoperative complications, and five patients (36%) presented with postoperative complications in the ARH group (Fisher exact test: 0.417). Table 4 and Additional file 1: Figure S1 shows the proportion of patients who presented with transoperative and serious postoperative complications (analysed together). In total, 36% (five) of the patients in the ARH group and 25% (four) of the patients in the LRH group presented with serious complications. The relative risk of presenting complications was 0.70, 95% CI (0.23–2.11), *P* = 0.694. No difference was found in the proportion of women who presented with transoperative or serious postoperative complications in this series.

**Table 4:** Transoperative and postoperative complications

**Variables**	**ARH (14)**	**LRH (16)**
Complication	5 (36%)	4 (25%)
No complication	8 (65%)	8 (75%)

ARH: Abdominal radical hysterectomy; LRH: Laparoscopic radical hysterectomy.

**Figure 2:**
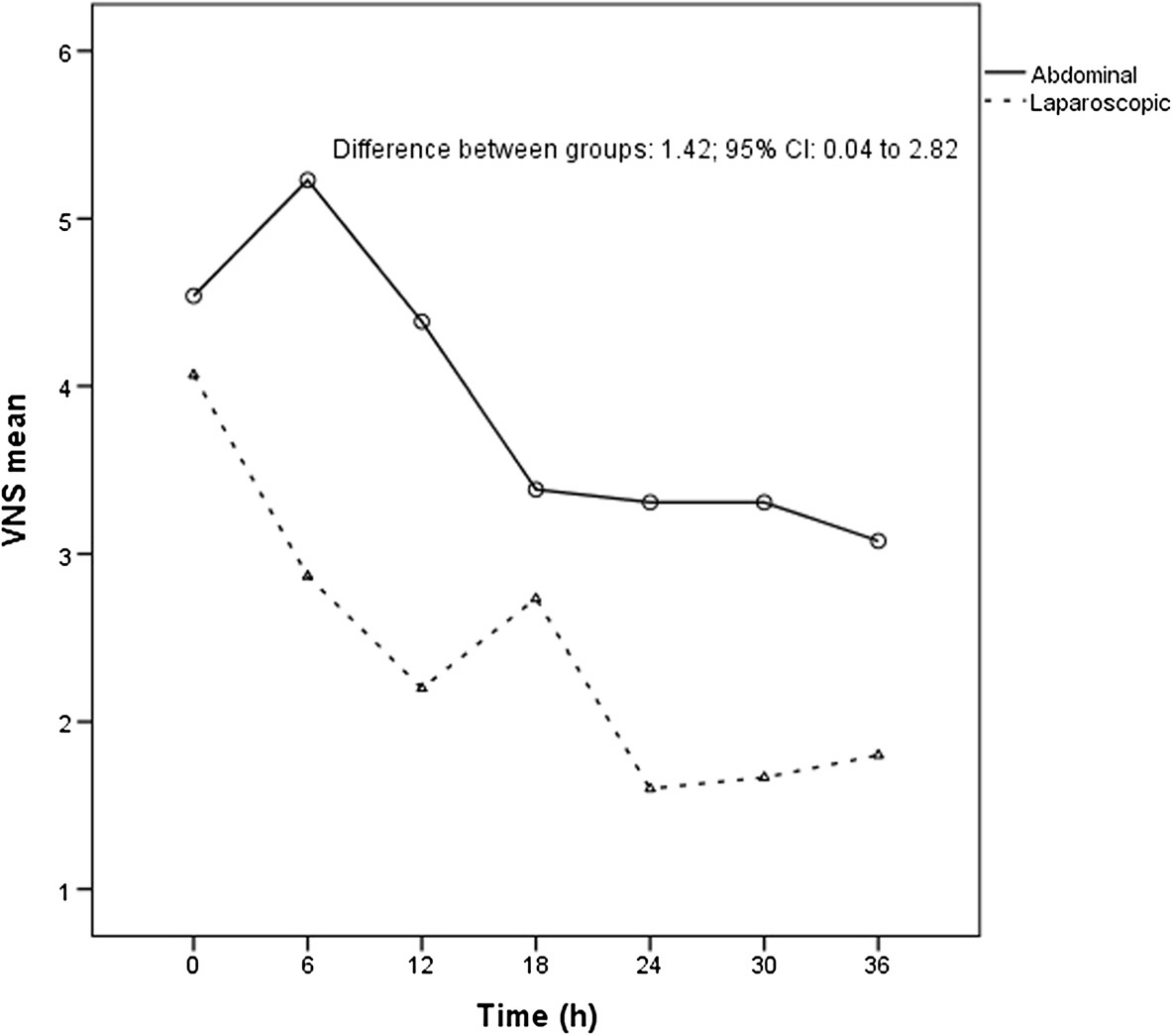
Postoperative pain scores 36 hours after surgery.

Figure 2 shows postoperative pain curves. LRH group mean pain score was significantly lower than ARH after 36 h of observation (*P* = 0.044; mean difference score: 1.42; 95% CI: 0.04–2.80) (Figure 2). In the LRH group, one patient (6%) requested “on demand” morphine; in the ARH group, two patients (15%) requested “on demand” morphine (Fisher exact test 0.586) (See also Additional file 2: Figure S2).

## Discussion

Laparoscopic radical hysterectomy had lower pain scores compared with abdominal radical hysterectomy in this series. Some studies comparing laparoscopic procedures in benign gynaecologic diseases with abdominal or vaginal procedures support our findings. A recent study randomised 41 patients who underwent laparoscopic hysterectomy (LH) and 41 patients who underwent vaginal hysterectomy (VH) for benign diseases. Postoperative pain was the primary endpoint and was measured using a 10-point verbal analogue scale (VAS). The LH group showed lower VAS pain scores than the VH group at 1, 3, 8, and 24 hours post-surgery [21]. A randomised study compared 19 patients who underwent laparoscopic myomectomy and 21 patients who underwent abdominal myomectomy. The pain scores were measured using a 10-point VAS. The laparoscopic myomectomy group had significantly lower pain scores than the abdominal myomectomy group at 48 h (2.1 ± 1.5 – 4.0 ± 2.3, *P* >0.01) and 72 h (1.1 ± 3.7 *P* >0.01) post-surgery [7]. A meta-analysis compared laparoscopy versus laparotomy in benign ovarian tumours. The odds of being pain free at 24–48 hours post-surgery were significantly greater for laparoscopy (110 patients) than laparotomy (108 patients) at 24–48 hours post-surgery [5]. VAS and NRS are considered equally accurate in assessing postoperative pain and superior to a four-point verbal categorical rating scale [22].

The differences in the evaluated pain scores can be explained as follows. First, laparoscopic surgery uses electrosurgical devices for dissection and coagulation, whereas abdominal procedures require clamps, scissors and tying knots, particularly when used in abdominal radical hysterectomy. Therefore, ARH like other open techniques, could cause a wider inflammatory reaction [23,24]. Second, wider incisions are subjected to more tension, which can lead to more pain [25]. The use of morphine “on demand” can alter the results, but we believe that this use did not interfere with our results, as there was no significant difference in the morphine request between the LRH and ARH groups.

To our knowledge, there is no randomised controlled trial published on laparoscopic radical hysterectomy versus abdominal radical hysterectomy that must be performed entirely by laparoscopic approach [26,27]. A randomized controlled trial comparing laparoscopically assisted vaginal radical hysterectomy and ARH was published, where the inferior part of parametrium and paracolpium were transected by vaginal approach [28]. This study included patients with tumours <2 cm and postoperative pain was measured by the amount of analgesic consumption [28]. Postoperative acute pain can be reliably measured by NRS and VAS, which are considered correlated [29]. They function best for the patient’s subjective feeling of present pain intensity. They may be used for worst, least, or average pain over last 24 h, or during the last week [22]. One of the strengths of the present study was the fact that assessment of present pain intensity as 5^th^ vital sign was performed every 6 hours.

The current study shows that LRH and ARH do not differ in terms of postoperative complications. Four patients presented with transoperative complications, and three of those presented with postoperative complications that could be consequences of the first complication presented. Studies of laparoscopic surgery for benign gynaecologic conditions show a lower rate of complications than abdominal procedures. A recent meta-analysis comparing abdominal, laparoscopic and vaginal hysterectomy showed that laparoscopic hysterectomy showed fewer wound or abdominal infections [25]. A comparative retrospective study comparing LRH (n = 35) with ARH (n = 54) described 18% vs. 53%, respectively, febrile complication rates (*P* = 0.001) [12]. A prospective study with retrospective controls compared 50 women submitted to LRH with 48 women submitted to ARH (controls); a comparative rate of urinary complications was observed in both groups [30]. Different surgical skill levels can decrease the performance of a surgical technique [31]. One limitation of our study was the fact that patient inclusion occurred during the learning curve of the leading surgeon; performing this trial at the beginning of the main surgeon’s training period most likely contributed to the LRH complication rate. A Finnish cohort of all hysterectomies performed in Finland from 1993 to 2005 reported a progressive decrease in serious complications for laparoscopic hysterectomies [32].

Oncological safety is always a concern in surgical studies. We included the parametrial extension measures to evaluate the surgical radicality, as proposed by Spirtos [8]. A retrospective comparative study comparing LRH (n = 34) with ARH (n = 37) found similar parametrial extensions: 3.8 cm (2.3–6.5) vs. 3.4 (1.7–7.0), *P* = 0.59; left parametrium 3.6 cm (2–6) vs. 3.5 (1.5–6.5), *P* = 0.82 [14]. A comparative retrospective study comparing LRH (n = 35) with ARH (n = 54) also found no differences in the parametrial extension [12]. Because we were not expecting any difference in the parametrial extension, we did not use any blinding. Longer parametrial extension measures for LRH could be explained by the magnification of tissue with the laparoscope, allowing a safer dissection near the bony pelvis.

## Conclusions

LRH presented lower pain scores after 36 h of observation in this series. The complication frequency was similar. Further studies are required to ascertain a comparable long-term survival rate.

## Abbreviations

AHS: Abdominal radical hysterectomy; LRH: Laparoscopic radical hysterectomy; NRS: Numerical rating scale; VAS: Verbal analogue scale; VH: Vaginal hysterectomy.

## Competing interests

The authors declare that they have no competing interests.

## Authors’ contributions

Conception and design: LSC, LFL, ATS, ANK. Study coordination: LSC. Inclusion and clinical data collection: LSC, LFL. Interpretation of data: LSC, LFL, ATS. Drafting and writing the manuscript: LSC, LFL. Revision of manuscript: LSC, LFL, ATS, ANK. All authors read and approved the final manuscript.

## Supplementary Material

Additional file 1: Figure S1 Transoperative and Postoperative complication. Click here for file

Additional file 2: Figure S2 Distribution of Pain Scores. Click here for file
