# Supplementary figures and images for: Postoperative pain and perioperative outcomes after laparoscopic radical hysterectomy and abdominal radical hysterectomy in patients with early cervical cancer: a randomised controlled trial
Source: Trials. 2013 Sep 12;14:293. doi: 10.1186/1745-6215-14-293 (PMC3856515; doi:10.1186/1745-6215-14-293)

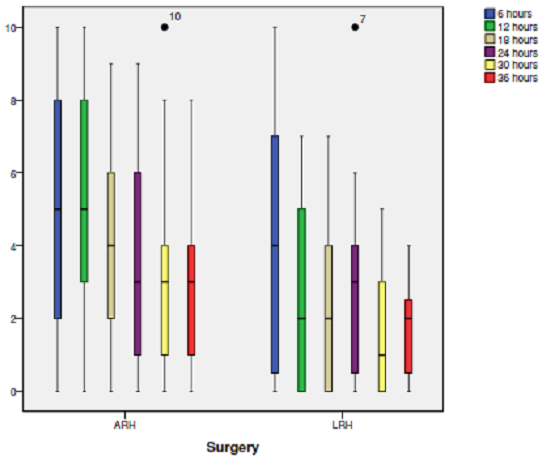

Supplement: Additional file 1: Figure S1 — Transoperative and Postoperative complication. [file 1745-6215-14-293-S1.tif]

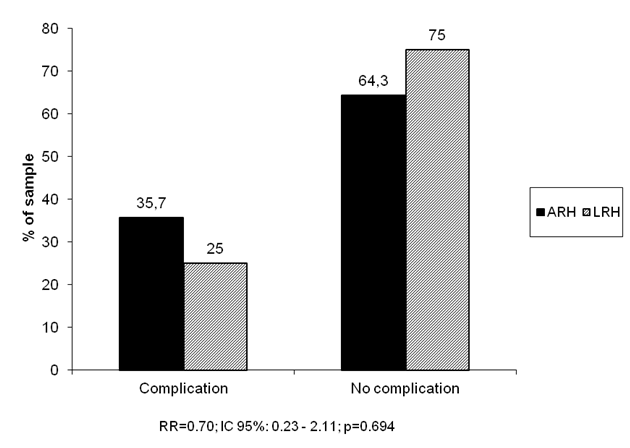

Supplement: Additional file 2: Figure S2 — Distribution of Pain Scores. [file 1745-6215-14-293-S2.tif]
